# Newly Diagnosed Hypokalemic Periodic Paralysis Triggered by COVID-19

**DOI:** 10.7759/cureus.47906

**Published:** 2023-10-29

**Authors:** Kelly Schulte, Maxwell Sheedy, Kavanya Feustel, Dmitriy Scherbak

**Affiliations:** 1 Internal Medicine, Sky Ridge Medical Center, Lone Tree, USA; 2 Internal Medicine, Rocky Vista University College of Osteopathic Medicine, Parker, USA; 3 Internal Medicine, HCA HealthONE, Denver, USA

**Keywords:** reversible cause of muscle weakness, bilateral upper limb weakness, bilateral lower limb weakness, sudden weakness, covid-19, familial hypokalemic periodic paralysis

## Abstract

Hypokalemic periodic paralysis (HypoPP) is a rare genetic disorder characterized by low potassium levels and episodic periods of muscle weakness. HypoPP has previously been attributed to numerous viral infections; however, cases related to coronavirus disease 2019 (COVID-19) are extremely limited. The current case is thus unique and involves a healthy 23-year-old male who presented to the emergency department after several uncharacteristic falls and three days of upper and lower extremity weakness. Initial labs revealed a potassium level of 1.1 mmol/L as well as being COVID-19 positive. Potassium supplementation helped stabilize his levels and relieved all of his symptoms. Based on an extensive clinical workup and significant family history of the mother and maternal grandmother with weakness in the setting of hypokalemia, a diagnosis of HypoPP was made. Upon discharge, he was placed on potassium-sparing diuretics to help prevent further symptom relapse and advised to complete genetic testing. With the high likelihood of the virus being endemic for years to come, clinicians should remember to consider HypoPP with patients with muscle weakness, especially in patients with concurrent COVID-19 infection, to minimize unnecessary workup and prevent potentially life-threatening symptoms of hypokalemia.

## Introduction

Hypokalemic periodic paralysis (HypoPP) is a rare neuromuscular disease that causes episodic paralysis in all extremities with predominance for lower limbs [1]. The degree of symptoms can be broad, ranging from painless paralysis to significant morbidity and a poor quality of life, with a prevalence of approximately 1:100,000 people [2]. HypoPP is often inherited in an autosomal dominant manner with the most commonly affecting the calcium channel, CACNA1S, and, less commonly, the sodium channel, SCN4A [2]. Recent studies support that alterations in calcium homeostasis from a CACNA1S mutation are likely linked to an altered function in the sarcolemmal adenosine triphosphate (ATP)-sensitive potassium (KATP) channel and thus cases of HypoPP are due to a secondary channelopathy [3].

Although a familial component is common, the disease manifestation has been reported with several etiologies with intracellular potassium shift being the overarching mechanism [3] The most reported precipitating factors are strenuous exercise, a high carbohydrate diet, stress, illnesses, and medications. These factors lead to a subsequent release of endogenous epinephrine and insulin, resulting in potassium shift intracellularly, an overall drop in serum potassium level, and the onset of paralysis symptoms [3]. Periodic paralysis has also been shown to be associated with severe hyperthyroidism and hypothyroidism [4,5]. We present a case of generalized extremity weakness in the setting of profound hypokalemia and concurrent coronavirus disease 2019 (COVID-19) infection.

## Case presentation

A 23-year-old African American male with no significant medical history presented to the emergency department (ED) with complaints of bilateral upper and lower extremity weakness for two days prior to admission. During that time, he reported difficulty standing upright and walking and had several uncharacteristic falls. Around noon on the day he arrived at the ED, he stated that he had to crawl out of bed to go to the bathroom due to extremity weakness, further described as “thigh tightness.” The patient was brought into the ED on a stretcher via emergency medical services. He denied any recent strenuous exercise, carbohydrate-dense meals, fasting, stress, or taking medications. He denied any respiratory difficulty or symptoms concerning for an upper respiratory infection. He had experienced similar symptoms previously, which would only occur two to three hours after a heavy workout and exposure to cold weather. The patient did note a previous hospitalization after his canoe flipped in water, resulting in hypothermia (93 °F), hypokalemia (3.1 mmol/L), and paralysis. The paralysis at that time resolved after replacing his potassium. His family history was significant for weakness and hypokalemia in his mother and maternal grandmother.

**Figure 1:**
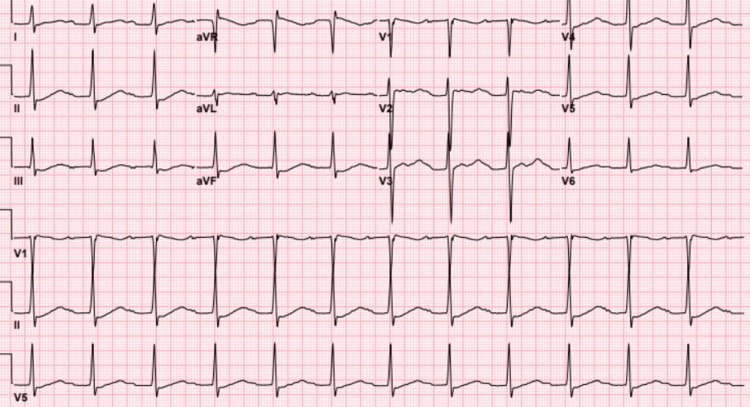
Initial EKG showing normal sinus rhythm, first-degree atrioventricular (AV) block, and flattened T waves.

Admission labs were significant for serum potassium of 1.1 mmol/L, as noted in Table 1. Magnesium levels at ED presentation were also unremarkable at 1.9 mg/dL, as seen in Table 2. Physical exam demonstrated 3/5 strength in all extremities with sensation intact, normal stature without hypertelorism, clinodactyly, or micrognathia. The electrocardiogram showed normal sinus rhythm with first-degree atrioventricular (AV) block and a prolonged QTc (568 ms) (Figure 1). Additional laboratory studies included thyroid stimulating hormone (TSH), free thyroxine (T4), and total triiodothyronine (T3), which all were within normal limits, as well as an incidental positive COVID-19 polymerase chain reaction (PCR) test. Urine studies showed a urine osmolality of 587 mOsm/Kg, creatinine of 91 µmol/L, sodium of 103 mEq/L, and potassium of 4 mEq/L.

**Table 1:** Potassium levels through hospital stay

Day and hour	Potassium levels (mmol/L)
Day 1	
Hour 0	1.1
Hour 3	1.5
Day 2	
Hour 7	2.1
Hour 10	4.1
Hour 14	5.4
Hour 17	4.5
Hour 19	5.2
Hour 21	4.5
Day 3	
Hour 28	4.6
Hour 30	4.8
Hour 33	4.5

**Table 2:** Magnesium levels through hospital stay

Day and hour	Magnesium levels (mg/dL)
Day 1	
Hour 0	1.9
Day 2	
Hour 10	2.8
Hour 14	2.6
Day 3	
Hour 30	2.4

Due to his severe hypokalemia and profound weakness, he was administered oral and intravenous potassium and magnesium replacement until the potassium levels normalized. His replacement began with 10 mEq potassium chloride (KCl), 2 g of magnesium sulfate, 800 magnesium oxide per os (PO) or by mouth, and another 120 mEq KCl PO shortly after. Over the next five hours, the patient received 120 mEq of KCl in three separate doses, 2 g of magnesium sulfate, and 40 mEq of KCl IV. During serial lab monitoring, his potassium levels uptrended to 5.4 mmol/L. Nephrology was worried about the upward trend continuing upwards and recommended preemptively lower potassium levels. To reduce the risk of arrhythmias from the hyperkalemia, he was subsequently treated with calcium gluconate, sodium zirconium cyclosilicate, normal saline with bicarbonate, insulin, and dextrose 50% water to achieve a consistent potassium level of 4.5 mmol/L. The patient’s symptoms resolved with potassium. He was discharged with a prescription for acetazolamide 250 mg twice daily and spironolactone 50 mg daily with close nephrology follow-up to complete genetic testing. As of the time of the report, the patient has not followed up for outpatient genetic testing, to our knowledge. The patient did return to the hospital four months later for similar symptoms after walking his dog. The patient stated that he had been doing well and avoiding triggers until two weeks before hospitalization when he was supposedly not taking his medications. 

## Discussion

HypoPP can be diagnosed clinically, but the gold standard is through genetic testing [6]. Our patient met the criteria for a clinical diagnosis based on the European Neuromuscular Center (ENMC) International Workshop of 2000 (Table 3) [7]. Specifically, with the two separate occasions of low potassium, history of weakness and hypokalemia in the family, duration of more than two hours, positive triggers which included hypothermia, strenuous exercise, viral infection, and improvement of potassium intake [2,3,7]. The last inclusion criterion is the absence of any other etiology for hypokalemia [2].

**Table 3:** HypoPP diagnosis criteria according to ENMC International Workshop, 2000 * Must satisfy all four criteria to meet diagnosis HypoPP: hypokalemic periodic paralysis; ENMC: European Neuromuscular Centre Reference: Stapleton, 2018 [7]

S. No.	Criteria*
1.	Two or more occurrences of muscle weakness with serum K < 3.5 mEq/L
2.	One attack of muscle weakness in the proband and one attack of weakness in relation to documented serum K < 3.5 mEq/L
3.	Three of the following six parameters: Onset in first or second decade, Attack duration > 2 hours, Positive triggers (carbohydrate-dense meal, after exercise, stress), Improvement with K supplement, Positive family history or genetically confirmed channelopathy, Positive McMannis short exercise test
4.	Exclusion of all other causes of hypokalemia

Thyrotoxic periodic paralysis, although uncommon, can present with proximal symmetrical lower limb weakness and can progress to involve all four limbs and the respiratory musculature [7,8]. However, a normal thyroid panel ruled this out in our patient. Anderson-Tawil syndrome (ATS) is another rare genetic disease with paralysis, cardiac arrhythmia, and distinct skeletal and facial abnormalities [2]. Our patient did not have any skeletal anomalies consistent with ATS. Ruling out other metabolic disturbances is essential to rule out any secondary causes of hypokalemia, including but not limited to gastrointestinal, renal, endocrine, and iatrogenic problems [9]. Our patient had a urinary potassium of 4 mEq/L, which would rule against any intrarenal potassium wasting disorders such as renal tubular acidosis or an increased aldosterone effect. The normal serum magnesium values help rule that out as a source of the problem. Finally, the extensive history and physical exam helped rule out the effects of diuretics, diet, or gastrointestinal disturbances.

His clinical presentation was consistent with HypoPP, likely triggered by the COVID-19 viral illness, despite being asymptomatic. Stress, such as from viral illness and fatigue, has been noted to be a precipitating factor for HypoPP [3,10-12]. Although findings and research on the effects of COVID-19 have been rapid and forthcoming, there are still very limited case reports of COVID-19 triggering HypoPP with similar findings and workup as seen in our presenting case [12,13]. Current research suggests that the ability of the novel coronavirus to bind the angiotensin-converting enzyme 2 leads to dysregulation of the renin-angiotensin-aldosterone system and subsequent hypokalemia [14]. Although the reports of COVID-19-triggered HypoPP are limited, there are many documented cases of other viral agents acting as a trigger, including dengue, chikungunya, and varicella zoster viral infections [10,11]. Although the specific link of HypoPP triggered by viral illness is not entirely understood, an endogenous release of epinephrine from viral stress is a commonly proposed mechanism [11].

Current treatment for HypoPP is based on normalizing serum potassium initially by oral administration of KCl at a rate of 0.5-1 mEq/Kg [9]. Replacement with oral supplementation is associated with fewer adverse side effects; however, intravenous potassium may be warranted in cases with severe weakness and EKG changes [9]. Continuous electrolyte and EKG monitoring and routine muscle strength assessments are paramount when administering potassium to avoid any unwanted and potentially deadly complications, including paresthesias, drowsiness, mental confusion, flaccid paralysis, and cardiac arrhythmias that could precipitate cardiac arrest if not caught [15]. Magnesium levels should also be evaluated when working up these patients due to their ability to increase potassium secretion in the distal tubule and collecting duct through the renal potassium secretory channel (ROMK) when magnesium levels are low [16]. By monitoring this, clinicians may also recognize and easily correct causes of prolonged QT and avoid potential fatal arrhythmias such as Torsade de Pointes [17].

Preventative treatment should include education of patients to avoid triggers and prescribe medications. Carbonic anhydrase inhibitors (CAIs) are not entirely understood but are thought to reduce the frequency of paralysis by increasing the opening of calcium-activated potassium channels [9]. Additionally, it is believed to reduce the accumulation of intracellular sodium, leading to a subsequent decrease in toxicity and muscle degeneration [2,9]. Acetazolamide 250 mg twice daily is an appropriate starting point with minor adjustments based on how the patient responds [7,9]. Prolonged treatment with acetazolamide may also lead to metabolic acidosis, reinforcing the need for routine laboratory monitoring with patients. Aldosterone antagonists such as spironolactone cause increased sodium and water excretion while sparing potassium [9]. Some authors suggest a starting dose of spironolactone of 25-100 mg daily is an appropriate first step for HypoPP, but caution awareness of documented adrenergic side effects and switching to triamterene [50-150 mg/day] or eplerenone [50-100 mg/day] for treatment [18]. 

## Conclusions

HypoPP is a potentially life-threatening medical emergency due to the patient's profoundly low potassium and subsequent risk for cardiac arrhythmia and respiratory insufficiency. Clinicians should have a high index of suspicion of electrolyte disorders such as HypoPP, especially when males in earlier decades of life present with generalized unexplained musculoskeletal weakness. When working up patients for HypoPP, clinicians should obtain an EKG, electrolyte levels, thyroid panel, urinary analysis, and a solid physical assessment to help rule out other similar causes of hypokalemia. Continually striving to include various differentials should improve recognition and the overall treatment and care for these patients.
